# How digital communications contribute to shaping the career paths of youth: a review study focused on farming as a career option

**DOI:** 10.1007/s10460-022-10335-0

**Published:** 2022-08-01

**Authors:** İlkay Unay-Gailhard, Mark A. Brennen

**Affiliations:** 1 UNESCO Community, Leadership, and Youth Development, The Pennsylvania State University (PSU), 307 Armsby Building, University Park, PA 16802 USA; 2 Leibniz Institute of Agricultural Development in Transition Economies (IAMO), Theodor-Lieser-Str. 2, 06120 Halle (Saale), Germany; 3 UNESCO Community, Leadership, and Youth Development, The Pennsylvania State University (PSU), 204C Ferguson Building, University Park, PA 16802 USA

**Keywords:** Literature review, Digital communications, Career, Youth, Rural life, Farming

## Abstract

Can the power of digital communications create opportunities for overcoming generational renewal problems on farms? This interdisciplinary review explores the reported impacts of digital communication on career initiation into farming from a global perspective via the lens of career theories. Seventy-three papers were synthesized into two domains: (1) the impact of digital communication interactions on farming career initiation, and (2) the dynamics of digital communication initiatives that create opportunities to inspire youth into farming. The finding shows that the mainstream literature primarily aims to support the continuity of farming careers but pay little attention to the potential of digital communication to attract youth into farming. This review argues that career communications for farming receives insufficient attention, and could be better integrated into agricultural communications strategies by using the potential of digital communications. Study concludes that while economic and geographic factors, as well as societal and cultural norms, lead to negative perceptions on farming careers, there are three pathways that may contribute to breaking down these negative perceptions. Firstly, taking the changing nature of career motivations, such as the trend towards sustainable farming linked to self-fulfillment, among today’s youth into consideration is essential. Secondly, highlighting technological advances in digital agriculture practices, like geographical flexibility or innovation capacity of farming, for example, is important to increase awareness about new opportunities in the profession. Lastly, communication campaigns with targeted groups (e.g., young females) play a role to change the negative perceptions of the rural way of life and the farming profession.

## Introduction

A lack of interest in farming among youth is a global phenomenon that has led to research and studies on concepts such as “generational renewal problems on farms” (Meuwissen et al. 2019), the “young farmer problem” (Eistrup et al. 2019; May et al. 2019), and the “greying of the farming population” (Conway et al. 2020). There are several reasons and factors that youth lack interest in farming as a profession. Apart from economic (e.g., high costs of land and machinery) and geographic (e.g., needing to be involved in a rural way of life) dimensions, this lack of interest in farming among youth is also strongly tied to societal and cultural norms.

These social and cultural norms may be summarized as follows:

(i) *an absent image of the farmer as a role model in society*: Youths’ subjective perceptions towards farming are often associated with difficult and dirty physical work, low levels of prestige, low income, no holidays, occurring only in rural areas and being a profession with limited innovation opportunities (Simões et al. 2021; Feinberg 2020; Accesstoland 2018; Yoon et al. 2021; Unay-Gailhard et al., 2019);

(ii) *emotional dimensions*: Different to other family businesses, for family farms emotional factors can play a strong role, such as having an emotional attachment to land, prioritizing personal or family values and wanting to be involved with farming that’s not necessarily profit-oriented (Lans et al. 2017);

(iii) *cognitive dimensions*: increasingly, children of farmers are showing interest in other careers (Accesstoland 2018). For farm children raised with uncertainty or tension surrounding the family farm business, such as uncertainty regarding farmland transfer or farmland access, competing visions among siblings, or questions surrounding equity in general, as well as family stress due to a lack of childcare availability in remote rural regions lead to pursuance of non-farm careers as adults (Rissing et al. 2021);

(iv) *gender dimensions*: like science and engineering, the farming profession shows gender disparities and is perceived as a male profession. There is a lack of farmer identity, and female farmers are often viewed as the “farmer’s wife” or “helpers” on family farms (Ball 2020). This identity issue transfers to a lack of “farmer and mother” role models for youth growing up on family farms (Unay-Gailhard and Simões 2022). When it comes to working on farms, psychological factors such as social recognition and inner satisfaction have been reported as low for young females relative to young males for a career choice of farm manager (Lehberger and Hirschauer 2016). Overall, these social and cultural norms result in a low willingness among young women to be potential farm successors or to choose farming as a career option, including farm manager roles (Shortall et al. 2020).

All of these highlight the intersection of human values orientation, technology, and behavioral outcomes. As we focus on the choice of occupation and how these choices overlay sociocultural conditions, the human value component can be seen as far outweighing simple economic dimensions.

In this review paper, by looking from a global perspective we explore how digital communication tools would be helpful for attracting a new, younger population into farming and create opportunities for overcoming generational renewal problems on farms.

As highlighted by digital communications studies (Madianou and Miller 2018; Schaffers et al. 2020), the use of digital media is not just changing technology levels, but it also results in changes to the relationship between human values, attitudes, and behavior, resulting in emotional, social, and moral consequences. The question of what impact this new relationship will have on initiating careers in farming is the motivation of this review.

This study contends that this lack of interest towards farming as a profession needs further attention through the use of communication campaigns and tools from both government and civic society (government agencies, agricultural extension networks, NGOs, farming and commodity organizations, etc.). These communications initiatives can help to overcome the negative perception towards the farming profession, to provide information on farming career opportunities, and most importantly initiate the choosing of farming as a career option.

The main purpose of this review is to provide a comprehensive, interdisciplinary integration of prior findings to synthesize existing knowledge and draw scholarly attention to the study of digital communications and farming career initiation. To achieve this goal, this review study defines and focuses on two research questions:

- **Q1:** What are the new and evolving forms of digital communication interactions that initiate careers in farming?
- **Q2**: In which ways may the dynamics formed through digital communication interactions be used to create communications content to initiate farming careers?

To guide the study in developing a review protocol, the PRISMA- Preferred Reporting Items for Systematic Reviews and Meta-Analysis Method (Moher et al. 2009) was followed with a focus on three objectives: (i) an inventory of digital communication tools used to support the initiation of farming careers; (ii) the key ideas and rationales that lie behind these tools; and, (iii) the reported impact of the role of digital communication tools to create career initiation.

In order to attract and recruit youth (coming from both farming and non-farming families) into farming, exploring their career development paths in the digital age is considered beneficial. Therefore, this study is based on recent career development theories, including career construction theory, chaos career theory, happenstance learning theory, and social cognitive career theory.

These theories incorporate three elements in career development in modern societies: (i) change (e.g., unstable situations with frequent changes in work-related tasks); (ii) chance (e.g., the unplanned and unpredictable nature of career choices); and (iii) constructiveness (e.g., the proactive nature of individuals with self-organizing and meaning-making tendencies, which help to build new competencies). To provide a more thorough analysis of the role of digital communication tools on the topic at hand, the results of this review study will be discussed within the lens of these recent career theories.

This article is structured as follows: Sect. 2 provides a theoretical background and introduces contemporary career theories that have informed this research. Section 3 describes the methodology and provides information about the data collection and clustering, while Sect. 4 presents the findings within two subsections and in the order of the two research questions. Section 5 discusses the results of the review study via recent career theories and ends with the remarks on future research that would be beneficial for expanding on this topic. Section 6 provides concluding remarques.

## Contemporary career theories informing this study

Career development refers to the process an individual undergoes to construct their occupational endeavors over a lifetime. Recent career development studies have suggested important elements that help us to understand the changing nature of contemporary careers in late modern society. Different than earlier career development studies that frame career motivations as planned and predictable, recent career studies consider the potential objective and subjective influences on individuals’ careers and include the effect of unplanned and unpredictable factors while explaining career choices.

The *chaos theory of careers* argues that an individual’s career development can be influenced more by outside factors than by any particular individual factors (Pryor and Bright 2014). The *social cognitive career theory* increases our understating on the relationship between social environments and career decision making (Lent et al. 1994). Our study is guided by the three elements of “change”, “chance”, and “constructiveness”, which have been used by recent career theory research to understand modern career construction paths.

Change: this element represents the dynamic, interactive, and adaptive nature of career development over the life course of an individual. Under *career construction theory*, how individuals manage to navigate through the changing career dynamics is explained by the concept of self-making, which includes elements of coherence and openness to change (Savickas 2005). Within these dynamics, reconstructing a career through self-making involves lifelong motivations and helps in the continuity of creating meaning out of new career choices and experiences.

Chance: career involvement may also include situational, accidental, and unintentional occurrences, thus being driven by unplanned and unpredictable events (e.g., being involved in a career without education or clear milestones in mind but because of economic situations). Chance events defined as significant influence the narratives of an individual’s career, and how a career subsequently develops (Rojewski 1999; Salomone and Slaney, 1981). The *chaos theory of careers* argues that career development has a consequence, which is the unpredictability of chance (Pryor and Bright 2014).

Constructiveness: this element encompasses the striving of individuals to construct or build meaningful perceptions to understand themselves in the world and learn from their experiences. *Happenstance learning theory* argues that both planned or unplanned events that individuals experience in their lifetime create learning experiences that impact their cognitions through the gaining of new competencies such as skills, interests, beliefs, and sensitivities (Krumboltz 2009).

The intersection between recent career development studies and youth studies provides guidance and helpful information on the younger generations’ career motivations. In the literature, these motivational factors are defined as “millennium traits”. In recent youth studies, millennium is often associated with *boundaryless*, (e.g., high levels of psychological adaptability and physical mobility), *protean* (e.g., the personal management ability of one’s career based on self-awareness) and *intrinsic* (e.g., engaging in a career with meaningful values) motivations (Boyle 2022).

In this study, the career development of young professionals has been viewed as a process instigated by psychological, cognitive and behavioral dimensions. When combined, the three modern society career development elements of change, chance and constructiveness are viewed as intercepting with younger generations’ career motivations.

## Methodology and description of data

**Fig. 1:**
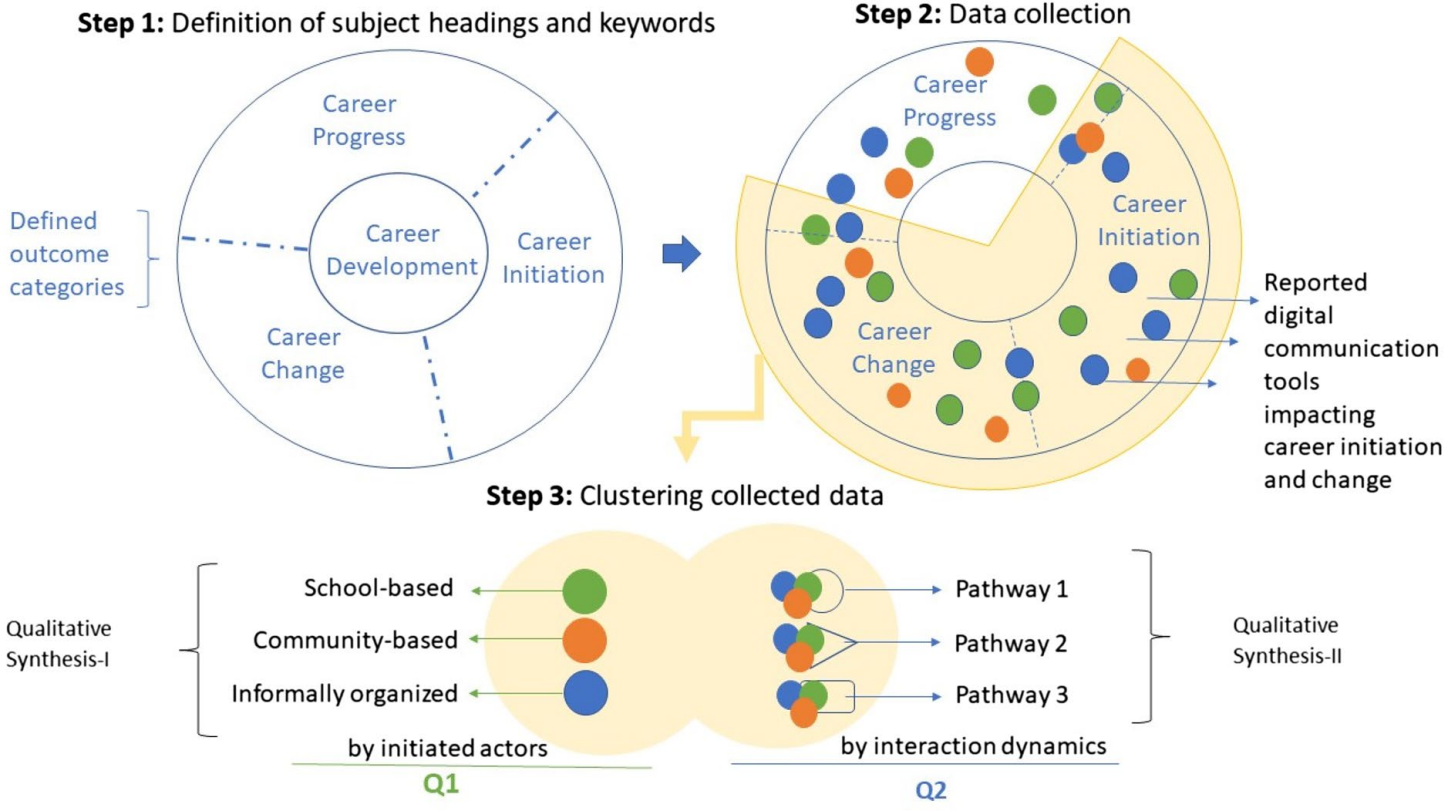
The three methodological steps of this review study

This review study on the relationship between digital communication tools and initiating a career in farming followed three methodological steps, which are summarized in Fig. 1.

### Step 1: Definitions of the subject headings and keywords

This literature review on the impact of digital communications on career initiation was conducted at the intersection of four fields: digital communications, vocational education (which mainly comprised of career development studies), youth studies, and agrarian literature (including rural sociology and farm entry studies).

Due to the limited number of studies at the intersection of all these fields, this review broadens the definition of career initiation outcome, and applies insights on the reported impacts of digital communications on career progress and career change. To relate the three career development pathways, the intersections between the categories of career “initiation”, “progress”, and “change” were coalesced for data clustering (Fig. 1).

After the conceptual relationships were formed, a broader question (in line with Q1, and Q2) with four subject headings were formulated: *“Is the use of digital communication tools (intervention) associated with career initiation, career change and/or career progress (outcome) among youths (population) in farming professions (condition of interest)?”.*

Through consultation with a research librarian specialist, and based on inspiration from recent review studies in the research topic at hand, including the fields of digital communications (Fernández-Planells et al. 2021), career development studies (Barhate and Dirani 2021), and agrarian literature (Dias and Rodrigues 2019), search keywords were defined for each subject heading, as presented in Table 1.

**Table 1:** Subject headings and keywords used in the review study

**Intervention**: digital communication tools; “social network site” OR “social media” OR “SNS” OR “online social network” OR “Facebook” OR “Twitter” OR “Instagram” OR “LinkedIn” OR “YouTube” OR “online forum” OR “social media” OR “social web” OR “ICTs” OR “websites” OR “blogs” OR “mobile phone” OR “tablet” OR “smartphones” OR “online interaction” OR “online communication” OR “technology” OR “online” OR “virtual” OR “computer*” OR “cyber” OR “digital”
**Outcome**: career initiation, career change and career progress; “career*” OR “job” OR “skill” OR “work” OR “employment” OR “vocation”
**Population:** youth; “young” OR “youth”
**Condition of interest**: farming profession; (agriculture* OR farm*) AND entrepreneur*

In this review for some keywords study used an asterisk * as a truncation symbol to find variant endings of the keywords in the database

### Step 2: Data collection

**Fig. 2:**
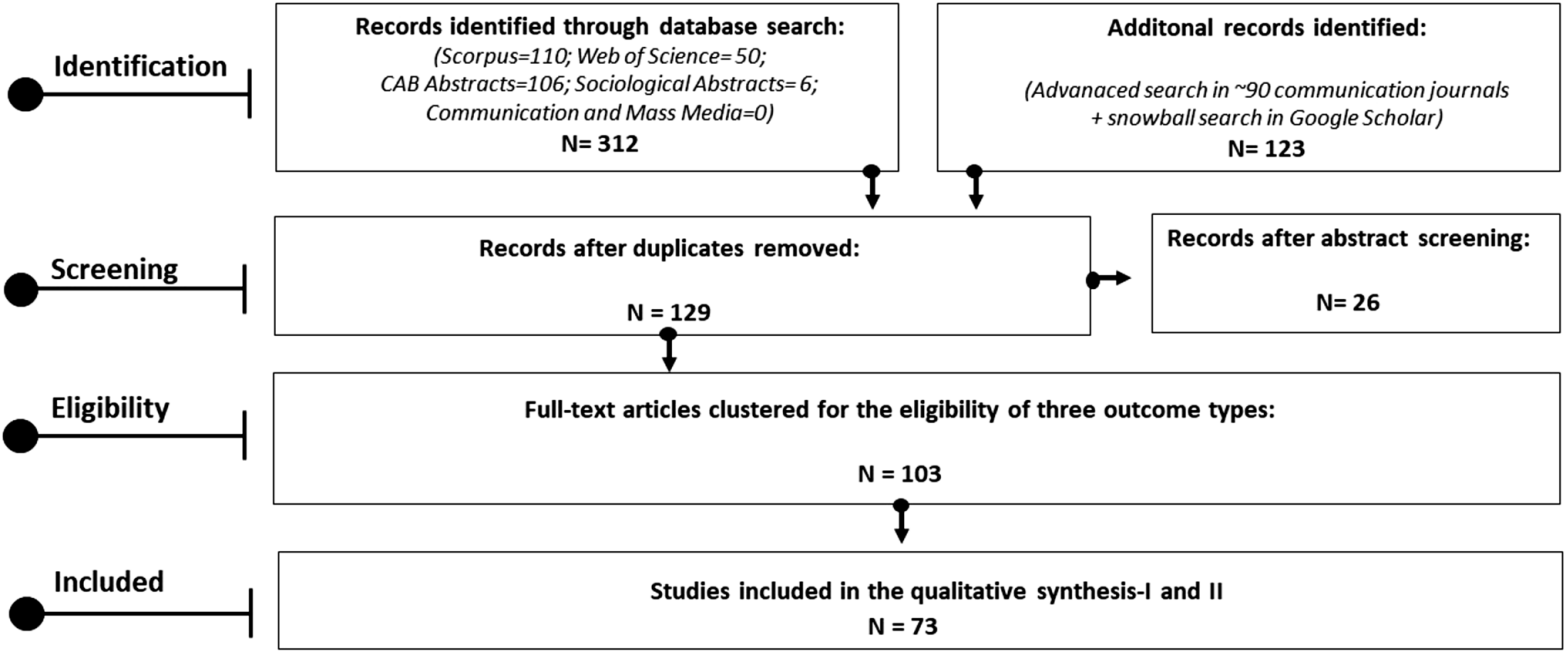
PRISMA Flow used in this review study

The data search strategy followed the PRISMA-method (Moher et al. 2009); the PRISMA Flow is presented in Fig. 2.

In the *identification* stage, searches were conducted using Scorpus, Web of Science, CAB Abstracts, Sociological Abstracts, and Communication and Mass Media Complete database. Additional searches were conducted for 94 communications journals in the Web of Science with keywords defined for the conditions of interest. This step was carried out since no articles were found in the Communication and Mass Media database. Lastly, by searching through references and citations, a snowball search method was applied to discover further articles using Google Scholar.

In the *screening* stage, duplicates were removed and in total 129 articles with abstracts were saved in Zotero. At the end of the abstract screening, 26 articles were excluded as they did not match the inclusion criteria, and 103 articles were considered to be worthy of full-text reading to determine whether they’d be eligible or not.

*Eligibility* decisions were made by clustering articles based on their corresponding results for the outcomes of “career progress”, “career initiation” and “career change”.

In the *inclusion* stage, the relevant studies were evaluated with three main inclusion criteria: (i) the research must contribute to the outcome of “career initiation” and/or “career change” (or the intersections among them) in the agriculture and farming sector; (ii) the research should focus on youth; and (iii) the study must have been published in the English language between the years 2012–2022. To approach two research questions, 73 articles were included for qualitative analyses I and II.

### Step 3: Clustering the collected data

For clustering the collected data, two methodological approaches for the two research questions were followed:

The qualitative synthesis-I: for Q1, the collected data was clustered by initiated actors, and clustering categories were based on the youth mentoring literature, which considers mentoring actors as formal (school-based, and community-based) and informal (parents, neighbors, political, religious, and media personalities).

The qualitative synthesis-II: for Q2, the collected data was clustered by the dynamics of digital communication interactions. The method of clustering the data into pathways followed the review study by Stevens et al. (2016) where the authors use pathway approach not as a passage in which the effects lead to outcomes, but a coalescence of activities that form a pattern in the way it implicates the outcome. In this step, the collection of studies was qualitatively analyzed in consideration of the various roles of digital communications in domains that overlap with agricultural communications topics (e.g., environmental communications, science communications). As a result, we identified three pathways of influence on career initiations.

Different than clustering by initiated actors (as for the qualitative synthesis-I), in this second stage (qualitative synthesis-II), we do not provide a detailed overview of the used communication tools (e.g., the impact of 3D virtual platforms in use in schools for career choices), but rather we highlight how the intersection of actors (users and providers) on the communication platforms brings about emergent phenomena as an opportunity for agricultural communications to initiate a farming career. Pathways allow us to illustrate the current dynamics being shaped by users and providers, and give contextualized interpretations.

Description of collected data: two main patterns.

The collected data suggests two main patterns. First, considering the growing attention to the link between digital communication applications and farming from very diverse disciplines, the impact of digital communication applications on “career progress” is highly established and guided by several literature reviews (Jimenez et al. 2021; Araújo et al. 2021; Sánchez et al. 2020; Klerkx et al. 2019). However, relative to “career progress”, there are few studies on the “career initiation” and “career change” effect of digital communication on the farming profession.

Second, even though review studies represent a diversity of social science perspectives from psychology to communication, three main social science disciplines of education, agribusiness management, and rural development mostly question the relationship between digital communications and career initiatives towards farming both in developing and developed countries.

## The qualitative syntheses results

The qualitative syntheses are detailed in two subsections below in order of the research questions. Table 2 provides a summary of the qualitative synthesis.

**Table 2:**
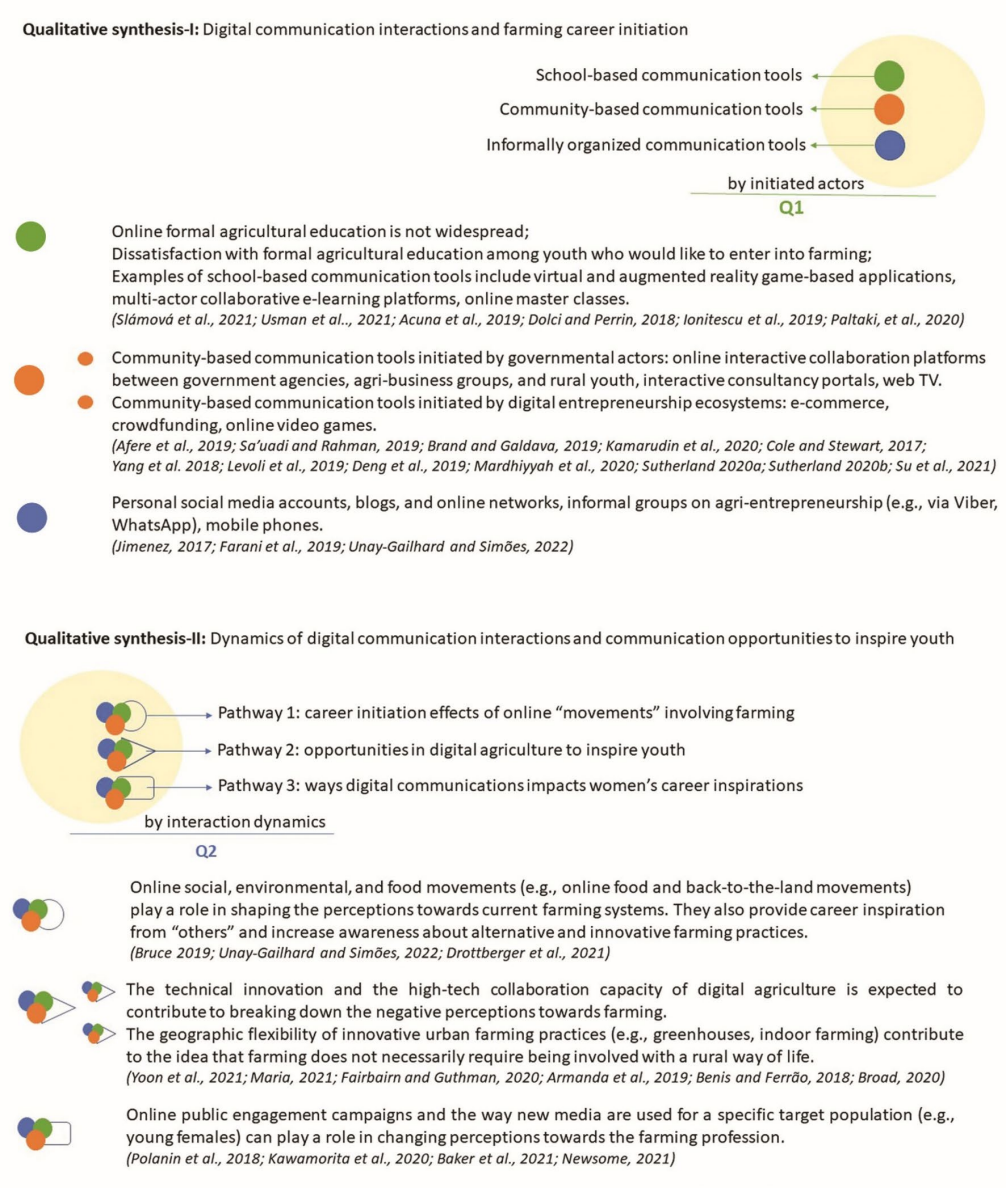
Summary of qualitative synthesis

(1) The provided summary results include the best-matched reported findings in terms of our study question, and details for each key summarized point are provided in the following section. The summarized points are ***bolded and italicized*** in the following manuscript for easy identification. (2) In this review study, “digital agriculture” refers to the digitalization of agricultural and food production systems, which includes several farm-related tasks managed by digital tools for both on-farm and off-farm activities

### Qualitative synthesis-I

The reviewed studies that report on the impacts of new and evolving forms of digital communication interactions on the categories of “career initiation” and “career change” are classified by the initiated actors’ structural forms (formal vs. informal), and presented in this subsection as: (i) school-based digital communication tools; (ii) community-based digital communication tools; and (iii) informally organized digital communication tools.

#### School-based digital communication tools

The school-based digital communication tools mainly used by formal education institutions comprise diverse tools such as Learning Management Systems (LMS) (e.g., free or paid online education platforms such as Massive Open Online Courses, MOOCs); Information and Communication Technologies (ICT) (e.g., internet-based applications like Zoom); publishing and formal education sharing tools (e.g., eBooks, podcasts); online collaborative systems (e.g., Google Docs); social networking platforms (e.g., Academia.edu); interpersonal online communication tools (e.g., email); 3D Virtuals (e.g., games, virtual labs); and online assessment systems (e.g., computer note taking) (see Pinto and Leite 2020 for a review).

The growth in the use of digital communication tools in schools offers new opportunities to assist youth not only in achieving instrumental goals (e.g., improving learning performance) but also with career developmental goals (e.g., promoting career aspirations). The reported impact of school-based digital communications on instrumental goals is well established in the education and social psychology literature (Arulchelvan and Yunus 2020; Burnette et al. 2013; Denton-Calabrese et al. 2021; Akcaoglu and Green 2019). These studies provide insights on the positive effects of school-based digital communications on the career initiations of rural youth via their empowerment and learning skills, such as creating self-confidence, growth mindsets, increasing community engagement, promoting career aspirations; and improving cognitive skills (see Flynn et al. 2022 for a review on rural youth).

Furthermore, the impact of school-based digital communications on career developmental goals has mostly been analyzed beyond a single profession perspective and with a focus on broader disciplines. Both of the studies by Hango et al. (2019) and Reid et al. (2016) report that school-based digital communication teaching tools increase career initiation towards STEM careers (Science, Technology, Engineering, and Mathematics) among rural youth. This is an important finding in the evidence that rural youth resulting low enrolments in STEM subjects relative to urban youth (Wilson et al. 2013). From a gender perspective, a study by Pinkard et al. (2017), shows that the use of digital communication platforms increases young women's interests in STEM career opportunities, a field typically characterized by gender disparities.

Even though many studies provide insights on the effect of school-based digital communications on initiation towards STEM careers, there is still unclarity about how digital communications influences initiation into a single career, and particularly a farming career. A review of the literature on online school-based agricultural educational initiatives reveals two important explanations for this uncertainty. The first is how ***the nature of online formal agricultural education is not widespread*.** A study by Slámová et al. (2021) on vocational education and training for multifunctional agriculture highlights that Open Education Repositories (OERs) based on online vocational education and training are still not widespread, resulting in a limited number of studies reporting on the impact of school-based communication tools on the initiation of farming careers.

The second reason is the ***dissatisfaction with formal agricultural education opportunities*** among youth that would like to enter into alternative farming careers. Research by Dolci and Perrin (2018) on neo-farmers (farmers migrating from urban to rural regions with the aim of being involved with agro-ecology) reports a high level of discontentment towards formal agricultural training, and highlights an increasing involvement with informal, online educational resources for gaining new skills and knowledge on farming. Even though new entrants are increasingly involved with unaccredited and informal training programs, as reflected by Accesstoland (2018), we still don't have sufficient findings on the demographics of this group and their career progressions into farming. As highlighted by Caskie (2018), the reasons for farmer lack of engagement in formal education and training are open to debate. The literature may benefit from further studies to assess the perceived value from online informal trainings among young farmers.

The recent literature provides insights into the link between evolving forms of digital communication interactions and career initiations towards farming via research by Usman et al. (2021) on youths’ tendencies to choose farming as a profession, as well as studies on augmented reality game-based applications (Acuña et al. 2019) and multi-actor collaborative e-learning platforms (Ionitescu et al. 2019; Oliveira and Cardoso 2020; Paltaki and Michailidis 2020).

Usman et al. (2021) argue that shifting career interests to under-represented professions like farming is difficult at the schooling age because parents’ and teachers’ formal career guidance has very little influence on adolescents’ career decisions. The authors report that around half of the students in secondary schools have not decided on a career path, and this “undecided” population can be only influenced through carefully chosen new communication tools. The authors suggest the use of the internet and social media among educational institutions to promote farming-related careers, proposing that digital media use is an efficient outlet due to the distal social environment’s (e.g., media, personalities in sports, politics) significant role on students’ career decision-making. Studies that highlight the role of the distal social environment on career decisions are further discussed in this paper within the informally organized digital communication tools section within the social cognitive career theory.

A study by Ionitescu et al. (2019) on ***virtual and augmented reality game-based applications*** for agricultural education provides initial insights on a 3D virtual teaching tool (Biz4Fun) developed for formal education. The tool includes games, videos and presentations about successful start-ups in agri-business and aims to be helpful for creating career inspiration for students in terms of developing and growing their business ideas within the farming profession. The authors contend that virtual teaching tools that include innovative educational materials and the custom designed social games makes agricultural education for young people more attractive, assertive and motivating.

In some cases, online formal and informal education overlaps and ***multi-actor collaborative e-learning platforms take shape***; participating actors come from many sectors including education, government, and agri-business.

For example, a study by Acuña et al. (2019) reports on ***online master classes*** in horticultural business that include the involvement of internationally recognized higher education institutions and entrepreneurs in the horticulture sector. The authors report that the majority of graduates of the online master classes intend to put their business plan into action, and the success of the online class relies on factors such as quick interaction among participants, international collaboration experience, short- and long-term networking opportunities, and feedback on business plans from industry and academic examiners.

Other exploratory case studies on digital ***e-learning and information sharing platforms*** (under the scope of EU-funded NEWFOOD and SPARKLE projects) describe a single project and give insights into how interpersonal digital communication tools could be used to create interest in farming among both university students and interested stakeholders through innovative food product ideas (Oliveira and Cardoso 2020) and providing knowledge on sustainable precision agriculture practices (Paltaki and Michailidis 2020). These case studies reveal that the effectiveness of digital e-learning platforms for business idea inspiration require not only ongoing trainings, but also the initiation of long-term collaboration among platform participants.

#### Community-based digital communication tools

The community-based digital communication tools include involvement of actors from formal institutions among government; civic society (government agencies, agricultural extension networks, NGOs, farm & commodity organizations); research institutions; and private businesses, most often taking the form of multi-actor collaborative platforms.

Different from school-based digital communication platforms, community-based digital communication platforms may differ with respect to target populations, structure, and their overall goals. For example, community-based digital communication goals may choose youth and adults to support their career construction paths both during and after their schooling periods. In terms of career support, community-based digital communication platforms’ goals range from preventing a school-leave, supporting a smooth school-to-work transition, and facilitating a job search, job access or career change (e.g., job-to-job transition).

Overall, findings show that the impact of community-based digital communication tools on career initiation into the farming sector have been studied more than school-based and interpersonal digital communication tools.

In this review, community-based digital communication tools are clustered along two lines of research. The first line (detailed as *Community-based digital communication tools initiated by governmental actors)* reports the impacts of agricultural-related governmental community-initiated platforms, which have mostly been studied in the context of developing countries within the social science disciplines of agriculture and rural development, youth studies, and information and communication technology.

The second line (detailed as *Community-based digital communication tools initiated by digital entrepreneurship ecosystems*) summarizes the impact of the digital entrepreneurship ecosystem (e.g., e-commerce and crowdfunding platforms) on career initiation and experiments with farming as a profession. This has been studied by multiple disciplines such as business, management and accounting, agribusiness management, geography, planning and development.

#### Community-based digital communication tools initiated by governmental actors

For this area of research, the literature to-date mostly focuses on implemented interactive online community-based projects with a high degree of involvement from government actors as initiators. This literature concentrates on the challenges in rural development, rural youth unemployment and agri-entrepreneurship domains, reporting positive implications of these projects for inspiring farming careers.

A study by Kamarudin et al. (2020) reports on the potential of ***an online interactive collaboration platforms*** in rural regions between government agencies and business groups in Malaysia and Indonesia, called as “creative village” approach. The authors show examples of new entrance populations moving into farming (e.g., organic production, medicinal plants and food production) initiated by changing consumer demands in urban regions.

Brand and Galdava (2019) present results for a “Digital Development for Feed the Future” project, which was comprised of ***online interactive consultancy*** and successfully implemented in Guatemala, Ghana, and Nepal. The authors highlight the potential opportunities of digital tools to shift perceptions towards farming, to teach agricultural skills, and to share agricultural knowledge, such as sharing high-quality videos on practical farm work.

Another two studies covering ***online consultancy initiatives*** reflect on the potential effects of digital communication platforms initiated by agricultural-related governmental departments to attract unemployed youth into farming by providing detailed local knowledge (e.g., administrative procedures for start-up ideas in agribusiness, existing consultancies, courses, events in the farming profession).

Concreate examples are a ***web TV*** entitled “Agribusiness TV” in Africa (Afere et al., 2019), and an online One-Stop Information Portal for Agri-Entrepreneurship in Malaysia (Sa’uadi and Rahman, 2019).

#### Community-based digital communication tools initiated by digital entrepreneurship ecosystems

In the second line, we consider the definition of digital entrepreneurship as “*a subcategory of entrepreneurship in which some or all of what would be physical in the traditional settings has been digitized based on the use of digital media and technologies*” (Hull et al. 2007, p. 293).

Studies on this topic are not yet well established, however they include important future research questions on the impact of digital entrepreneurship platforms on agri-entrepreneurship initiatives. The main reviewed studies in this area have focused on digital entrepreneurship ecosystems and their roles for (i) increasing awareness of small businesses in the farming sector and (ii) the creation of new agri-entrepreneurship ideas by providing guidance and support to launch business plans.

Recent studies on the use of ***e-commerce**** and ****crowdfunding*** provide initial insights on the indirect effects of awareness and knowledge building through digital entrepreneurship platforms on career experimentation in the agriculture and farming sector. Both the findings by Su et al. (2021) and Yan et al. (2018) display that knowledge on e-commerce tools have an indirect effect on rural youths’ engagement with farming (with new types of agricultural business entities) as a profession in rural China. Furthermore, two studies have also investigated the use of crowdfunding platforms for the agri-business sector. A study by Mardhiyyah et al. (2020) deals with the attractive personal asset dimension of crowdfunding platforms for new agri-business startups. The authors report that the project period, risk and field are factors that influence people’s participation in agricultural business investments through crowdfunding schemes in Indonesia. An article by Filimonova et al. (2019) investigating two popular Russian crowdfunding platforms reports how crowdfunding platforms are alternative sources for financing new ideas in the farming sector.

Taking a broader perspective, there are also studies reporting empirical findings on the positive effects of having ***access to digital infrastructure*** on farm entry decisions for individuals with innovative farming ideas. An article by Levoli et al. (2019) reports on three case study results in Italy as proof of how the existence of a digital infrastructure attracts highly educated young agri-entrepreneurs and promotes social innovation. Furthermore, the digital infrastructure was found to be helpful for overcoming the disadvantages of remote rural regions that experience physical, social and cultural isolation. For the case of China, Deng et al. (2019) analyze the relationship between the use of digital communication tools (using a dummy variable of having household internet or not) and land abandonment, finding that internet use helps reduce farm households’ cropland abandonment.

The studies by Sutherland (2020a), (2020b) and Cole and Stewart (2017) are three of a few bodies of research investigating popular ***online video games*** based on farming and farming settings (e.g., Farming Simulator, Stardew Valley, and Hay Day) that are used for leisure and not in professional contexts with a specific skill development purpose. The authors highlight the power of video games in producing particular forms of idyllic rural utopia and influencing thoughts and feelings associated with farming, such as the development of empathy regarding the exploitation of real non-human animals (Cole and Stewart 2017), and reinforcing ideas such as low input use for farming (Sutherland 2020a). These types of studies would also benefit from future empirical research into the ways in which these online games impact career initiations in rural regions.

Lastly, the potential effects of formally initiated digital communications tools (as detailed within the divisions of school-based and community-based tools) on the career initiation of youth finds its rationale from career theories. Recent career development theories suggest a trend where individuals advance in their career paths by following personal values, such as self-fulfillment in their career path, rather than organizational values (Hall 1996). Today’s careers are highly tied to multiple meaningful values (*intrinsic*), such as maintaining an authentic sense of self or searching for a work-life balance (Mainiero and Sullivan 2006), and show *boundaryless* and *protean* motivations that reflect the unplanned/unpredictable nature of careers (Modestino et al. 2019).

This review holds that this shift in career construction paths is supported by digital communication technologies. Overall, the role of digital communication tools, notably the use of the internet and mobile devices in formal education, training and networking, is leading to changes in the career initiations of youth. The use of online formal education and networking platforms not only helps to improve career-related skills, but it also brings about opportunities to easily integrate knowledge from diverse sources, and reskill them for new, or unplanned/ unpredictable career plans that are in line with one’s career values.

#### Informally organized digital communication tools

In addition to formally initiated digital communication tools, career inspiration may arise through informal online social networking tools (e.g., blogs, personal social media accounts, social networks through platforms like Facebook or WhatsApp).

The role of informally organized digital communication tools on career decisions is mainly supported by empirical findings from the career development literature pointing to significant positive roles of distal social environment (e.g., media) on career decision-making (Bright et al. 2005).

In the agri-business literature, the use of social media seems to play an important role among actors in the farming sector through the linking of people and ideas (see Bowen and Morris 2019 for a review). Furthermore, online informal networks (e.g., vegan organic networks) are most of the time involved with information sharing initiatives via websites or YouTube videos, with these information sources often being cited as important resources for learning farming practices online (Seymour and Utter 2021).

Nevertheless, with only a few studies, empirical studies focused on how informally organized digital communication tools impact youth career development in farming are still very limited. Moreover, rather than separating the two, studies that have investigated the effects of digital communications on career choices often group informally organized digital communication tools together with community-based digital communication tools.

The article by Unay-Gailhard and Simões (2022) provides some insights on the impact of digital communication tools for choosing farming as a career. Based on narrative interviews with young farmers in Greece and Portugal, the authors report how several ***personal social media accounts and blogs*** (e.g., a mushroom grower’s blog, a greenhouse farmer’s personal social media accounts, an ecological farming Facebook group) were viewed as positive sources of inspiration from others that directly or indirectly influenced career orientation towards farming.

Based on a descriptive-correlational analysis investigating group statements, Yaghoubi Farani et al. (2019) examine how membership in ***informal groups*** for agri-entrepreneurship knowledge exchange (via Viber and WhatsApp) effect agricultural students agri-entrepreneurial thinking in Iran. The authors report how these online groups had both positive direct and indirect effects, such as decisions on entrepreneurial actions and better entrepreneurial thoughts.

An ethnographic study by Jimenez (2017) on the effect of ***mobile phone*** usage among Mexican migrant farm workers in California explores mobile phone usage among the migrants themselves, and provide interesting insights on their non-migrant relatives' career decisions in the farming sector in the US. They conclude that, as the evolution of communication tools parallels the increasing migration from home countries, opportunities to find better working conditions are created, as well as decreased anxiety and unpredictability surrounding job searches on farms.

Finally, the social cognitive career theory (Lent et al. 1994), which is used to understand the relationship between social environments and career decision making, may be informative in understanding the role of online informal communication tools in providing information and inspiration, as well as self-efficacy beliefs that influence career choices. Social cognitive career theory proposes that social models (e.g., observing “others” who show similarities with one’s personal career values) reinforce messages to which one is exposed, and may affect one’s self-efficacy beliefs in certain careers (Lent et al. 2000).

Based on social cognitive career theory, this review study assumes that youth are likely to become interested in a farming career when they display strong self-efficacy through both finding information from and being inspired by “others” via online platforms. At that point, gaining the necessary skills and gathering social environmental supports would be important for carrying their career interests towards career involvement.

### Qualitative synthesis-II

By reviewing the dynamics formed by digital communication interactions in the domain of career development in farming, we identified three pathways. These pathways provide insights on the agricultural communication opportunities that can inspire youth to take up a career in farming.

#### Pathway 1: career initiation effects of online “movements” involving farming

The social, environmental and food movements (e.g., fair trade, local, organic, slow food, other gastronomic movements) play a strong role in shaping the perceptions towards our current agriculture and farming systems, in both positive and negative ways. Overall these “movements” mainly support the idea that industrial agriculture and food systems require urgent policy actions addressing the high costs that society pays for social, environmental, health, animal welfare, and gastronomic challenges (Stevens et al. 2016).

Within the literature in recent years there are an increasing number of studies providing insights on the impact of “movement” engagement (although they haven’t analyzed offline versus online movements) on the involvement with alternative farming practices. Within the urban agriculture literature, the socio-economic behavioral impacts of food movements are reported to facilitate collaboration, innovation and socio-economic organization, such as for community-shared gardens, food-sharing and urban food systems (Hearn et al. 2014). Seymour and Utter’s (2021) findings displaying that, most of the time, vegan farmers identify a personal or political commitment to the veganism movement as a primary reason for their veganic approach to farming. An article by Dolci and Perrin (2018) explores the impact of the back-to-the-land movement on neo-farmers and their strong emotional motivations that are reflected in both their farming methods and their changing way of life in rural regions. Stringer et al. (2020) also looks at farmers involved with "movements" centered on farming practices (e.g., slow food, gastronomic movements, artisanal farming) and reports an increased market share of these types of farming in recent years. An article by Farrell et al. (2022) reflects on the opportunity of the organic movement to encourage the next generation of young farmers to commit to the family farm and consider organic farming as a long-term career goal.

Even though these studies provide insights on the inspirational effects of being involved with “movements” on career initiation, there are still many uncertainties about how online “movements” potentially lead to farming careers. A review of the literature on digital "movement" initiatives provides a number of key findings, with three articles in particular advancing our understanding of the impact of online food movements, back-to-the-land movements (Bruce 2019; Unay-Gailhard and Simões 2022), and online agro-ecological environmentalist groups (Drottberger et al. 2021) on career initiation and change.

In the context of Europe, a study by Unay-Gailhard and Simões (2022) on two EU islands (Crete and the Azores) reports that involvement with ***online food and back-to-the-land movements*** creates awareness and sensitivity about certain topics, provides career inspirations and leads to involvement with alternative or innovative farming practices (e.g., getting involved with growing microgreens after a long discussion with a blogger). The authors argue that digital communication tools are helpful tools for linking youth career choices with social and/or environmental ideologies to reach career self-realization.

A study by Drottberger et al. (2021) explores the motivations and capacity building among young Swedish market gardeners who are attracting attention through sustainable lifestyle ideologies which are prompted by demand for healthy and locally produced food. The authors find that the values of the interviewed market gardeners are in-line with the global ***agroecology movement*** being facilitated by digital platforms.

A study by Bruce (2019) investigates the catalyzing role of organic food movements on the involvement into farming career in the US. Based on interviews with farm managers involved with alternative farming from non-farming backgrounds (referred to as “greenhorn farms” in their research), the authors report how food movement networks are supporting a new generation of farmers by providing new training opportunities and markets, as well as promoting alternative farmer role models in the new media.

#### Pathway 2: opportunities in digital agriculture to inspire youth

The digital agriculture that’s a part of our lives is an outcome of the fourth industrial revolution (e.g., the use of artificial intelligence, the “Internet of Things”, bioengineering technologies) and is expressed using different terms in the literature, such as “smart farming”, “precision farming”, and “agriculture 4.0” (see Klerkx et al. 2019 for a review).

In this study, digital agriculture refers to the digitalization of agricultural and food production systems, which includes several farm-related tasks managed by digital tools for both on-farm activities (e.g., online management programs, drones, satellites to monitor farm activities, the use of new technologies such as cellular agriculture to grow meat, dairy, eggs, etc.) and off-farm activities (e.g., use digital tools for data interpretations and to facilitate food value chains, etc.) (Klerkx et al. 2019).

Efficient communications about career opportunities in digital agriculture (e.g., through online agricultural, environmental and science communications, and public engagement campaigns) and its potential to result in career inspiration is a topic of interest in recent conceptual studies. In the social science literature, researchers have investigated the question of to what degree technologization would make the farming profession attractive (Daum et al. 2022). However, this topic has not been studied frequently enough to report on with empirical findings from a career communications angle.

Our review study documents two main motivational factors that are linked to the characteristics of digital agriculture practices and expected to make farming an attractive career option among youth. First is ***technical innovation and the high-tech collaboration capacity*** of digital agriculture. A conceptual study by Yoon et al. (2021) suggests that advances in digital agriculture have a high potential to attract youth, particularly those demonstrating strong interests in the science and engineering study disciplines. Successful online science communications campaigns for digital farming practices contribute to breaking down the negative perceptions, such as that farming requires low-skilled individuals and that it contains low levels of innovation opportunities. The rise in skilled worker demand in digital agriculture has been argued to show potential for involving white-collared farmers into the sector (Donnelly 2014), and providing collaborations with IT experts, advisors and agri-tech actors (Maria et al. 2021). Supporting these suggestions, an opinion report by Fairbairn and Guthman (2020) provides insights into how the ongoing COVID 19 pandemic is attracting new investors into digital agriculture because of food safety and security concerns.

Second is the ***geographical dimension*** of digital agriculture. As pointed out by Armanda et al. (2019) and Benis and Ferrão (2018), innovative and commercial urban farming practices (e.g., greenhouses, indoor farming) may let youth pursue farming careers not solely in rural regions where they may face land access problems. This geographic flexibility of innovative urban agriculture is reported to be seen as attractive to youth who have the perception that a higher quality of life only exists in careers outside the rural community. Furthermore, a study by Broad (2020) investigates a US-based indoor urban farming company that specializes in hydroponically grown herbs, and the effect of the company’s public engagement campaign and training initiatives for youth (call as “Next-Gen Farmers” training program). Accordingly, an article by Manning (2019) reports on the inspiring impact of the “Next-Gen Farmers” training program and its success for creating career initiation among youth as founders of indoor urban farming companies.

#### Pathway 3: ways digital communications impact women’s career inspirations

Another specific aspect of the effects of digital communications in terms of entry into farming is that of gender dimensions. Although there is a solid amount of background studies explaining the low involvement of females in the farming profession because of societal, cultural norms and financial and farm structural barriers (Shortall et al. 2020; Saugeres 2002; Ong and Lioa 2020; Pilgeram and Amos 2015), there is a growing body of literature focused on the switching of career identities from “farmer’s wife” to “farmer” among the new generation (Ball 2020; Perez et al. 2020; Tsiaousi and Partalidou 2020; Adro and Franco 2020; Unay-Gailhard and Bojnec 2021).

Our review study applies insights from agri-entrepreneurship, communication and rural sociology literature in order to delineate the ways through which digital communication interventions in rural regions support the inclusion of young females in farming careers.

Studies investigating the link between media and entrepreneurship in rural regions are an important stream of research (Gul and Demiryürek, 2020; Kawamorita et al. 2020). Because of the influential power of media on the behavior of rural society, it has the potential to create a positive image and support rural women entrepreneurs by providing information, improving skills (e.g., technical adaptability, work-life balance, network building), and disseminating success stories.

For example, following the information asymmetry framework, a study by Kawamorita et al. (2020) investigates the effect of ***new media (internet, mobile and social media)*** on the involvement of rural women in farming professions with a comparative perspective of Turkey and Japan. The authors’ findings suggest that access to media helps individuals understand the current market needs, and provides them with opportunities to respond to these needs with agri-entrepreneurship thinking.

With a case study in the USA, Polanin et al. (2017) provide insights on the Annie’s Project, which is a non-governmental organization that aims to empower women in the farming profession by using ***e-learning tools*** (e.g., videos, social media and a website). The authors results provide a better understanding of the positive effect of digital communication tools on the career initiatives of the various profiles of females living on farms, and how it’s helpful to create long-term on-farm business plans for both women farmers as well as women with off-farm jobs who live on farms, enabling them to change careers to farm businesses as a full-time job.

With another case study in Australia, the study by Baker et al. (2021) reflects on the effects of a ***public engagement campaign*** (called the “Invisible Farmer Project”) that aims to disseminate social media accounts of women farmers documenting their daily work, abilities, and rural perspectives. The authors explore how the project was powerful in shaping the perceived farming identities among participating female farmers, as well as engaged followers. The authors discussion displays how the use of social media may influence the career inspirations of future generations, seeing it as one of the important tools that may bring change to the masculine farming sector, which is characterized by invisibility, marginalization, and discrimination of female involvement.

This conclusion is also echoed by a study by Newsome (2021) looking into female farmers’ involvement with sustainable agricultural practices in Australia and confirming a positive effect of ***social media*** on career initiations. Among the narrative interviews, several participants highlighted how social media enables them to imagine alternative worlds and career opportunities in sustainable farming and helps them to perceive farming as a long-term career option due to financial (e.g., customer access) and social (e.g., building trust with sharing posts on daily work in farms) opportunities facilitated by digital communication tools.

## Discussion and directions for future research

Can the power of digital communications create opportunities for the challenge of how farming is viewed as a career option and to what extent would this be helpful for overcoming the problem of the generational renewal of farms? This interdisciplinary review study explores the evolving digital communications initiatives and their reported impact on career initiation towards farming from a global perspective.

Overall, this study explores the increasing interests in the relationship between digital communications and the image challenges faced by a rural way of life, although this field would benefit from a narrowed down focus on the negative perception towards the farming career among both urban and rural youth. This review study concludes with three main points to give directions for future research.

First, in terms of the number of studies, there is significant unequal distribution in the amount of research reporting on the impact of digital communications on career initiation relative to career progress in farming. The number of researched data for this review shows that the number of studies that make insights available on the relationship between digital communications and career initiation is quite low in comparison to those reporting on the relationship between digital communications and career progress. This finding shows that the mainstream literature on the digitalization aspects of agriculture primarily aim to support a continuity of careers for those who’ve already entered into farming, but pay little attention to the potential of digital communication initiatives to attract new entrance farmers, particularly among younger generations.

This scientific interest in the potential of digital transformation for career progress can partly be explained by how digital agriculture is understood in policy discourses to show a prioritization of maximizing food output (Lajoie-O'Malley et al., 2020) and the application of digital agricultural technologies within the agri-food system that’s mainly based on productivity (Prause et al. 2020). As concluded by the review study of Hackfort (2020), a productivist strategy is the current model in the ongoing digital transformation in the agri-food sector.

Second, our review suggests that the messages of new media and reported scientific results differ when it comes to how newcomers to farming are portrayed. Within the new media we observe a public image of new farmers that is different from previous generations. For example, both in the USA and in the EU, the media often depicts new farmers as young, including a good share of women; coming from urban regions with diverse socio-economic backgrounds; highly educated (with education in something other than agriculture a lot of the times); having no previous farming experiences, and often being career changers (see Dewey 2017; Raftery 2011; Roman-Alcalá 2013 for the USA, and Agroop 2018; Accesstoland 2018; Lenzi 2021 for the EU).

This new public image of entry into the farming profession provides insights about rising interests. However, in terms of age, there are still individuals who get involved with farming careers later in life or avoid being involved with full-time farming early in their careers. The career opportunity identification capacity of individuals is very low, particularly during the school-to-work transition period (Pindado et al. 2018). In the EU, individuals who are 40 years and older consider farming as a second career option (Pindado et al. 2018), and in the USA, more than one third of first-generation farmers are over 55 years old (Ahearn and Newton 2009; Bruce 2019).

Our review study captured the newcomers (those showing different backgrounds from previous generations) mostly within the “career change” category. Entry into farming careers was observed to have occurred from different occupations and those not necessarily related to the agriculture and food sector, such as chef, computer programmer, and international aid worker (Drottberger et al. 2021; Bruce 2019), with university-level education in disciplines other than agriculture, such as economics, mechanical engineering, forest engineering, tourism, sociology, geography, electronics, and informatics (Unay-Gailhard and Simões 2022). There are also studies confirming the new media definition of new entrants into farming with immigration to rural regions from urban regions (Dolci and Perrin 2018; Guarín et al. 2020; Unay-Gailhard and Simões 2022). However, the literature still lacks systematic studies that contribute insights on the demographic profiles of these newcomers, particularly for those in the younger age categories who are passionate about farming despite the negative images of this profession in the society.

Third, theoretical discussions, particularly elements of “change”, “chance”, and “constructiveness” highlighted in the recent career theories are important for understanding today’s youths’ career construction paths. However, regarding the theoretical backgrounds of reviewed studies, those exploring the changing nature of farm entry are less commonly based on theories to conceptualize this phenomenon.

As discussed by recent studies, theoretical models that are used in the previous agrarian studies to explain the farm entry decisions no longer identify the strategies currently utilized by today’s youth for career construction into farming (Bruce 2019; Unay-Gailhard and Simões 2022). Recent farm entry studies provide empirical findings on the changing nature of farm involvement as a career option that’s different from traditional farm succession within different pathways (Bruce 2019; Góngora et al. 2019; Stringer et al. 2020; Guarín et al. 2020). Therefore, using lenses of recent career theories is considered beneficial for explaining the changing nature of farm entry, and the role played by digital communications in the career construction paths among youth.

For example, considering the element of “change” (the adaptive nature of career development that includes the continuity of making meaning out of new career choices), our review provides two supportive explanations: the potential of digital communication tools to reskill individuals for new career choices and to enable them to create a professional identity for experimentation and for stability in this new career situation (e.g., farmers who’ve migrated from urban to rural regions and are involved with informal, alternative educational resources for gaining new skills).

Regarding the element of “chance” (having a career impacted by unintended situations driven by unpredictable events), the supportive role of digital communications, such as low cost and flexibility in terms of scheduling online educational resources and the potential to network quickly with individuals from similar or different backgrounds provides opportunities for new careers. These supportive factors gain importance when an individual is faced with chance events, such as unexpected financial crises, or personal events such as an unexpected job loss (e.g., involvement with innovative farming practices as a farm successor due to economic crises that influence the job opportunities in the urban labor market).

Finally, for the element of “constructiveness” (the quality of individuals to build meaning from life experiences, which is helpful for navigating both planned or unplanned careers), a powerful role of digital communications is observed through its visual (e.g., create role models) and emotional (e.g., emotional attachment to a certain way of life) ability to provide not only skills but also beliefs, interests, and knowledge on new topics (e.g., individuals who end up in a farming career by finding personal accomplishment and self-fulfillment in the agro-ecological movement).

## Conclusion

This paper contributes to two main challenges within the contemporary socio-economic research field: (i) the global difficulty of attracting youth into the under-represented profession of farming, and (ii) the growing pressure on institutions to respond to the demand for efficient youth- and career-focused communications in the digital age. These two challenges are particularly relevant for an interdisciplinary review study on the “impact of digital communications on initiating farming careers”, as this intersects several social science disciplines, namely: economics (e.g., agrarian literature, agri-entrepreneurship studies), politics (e.g., youth policies, generational renewal policies), psychology (e.g., vocational education, career development studies), geography (e.g., land abandonment), and sociology (e.g., rural sociology, digital sociology).

This study findings give that online career communications for farming receives insufficient attention, and could be better integrated into agricultural communications strategies (through online agricultural, environmental and science communications, and public engagement campaigns) by using the potential of digital communications. Our findings have practical applications for agricultural extension and rural communications specialists, as well as education institutions.

There are three online career communication strategies that could serve as pathways and contribute to breaking down the negative perceptions of the farming profession and create career initiation in farming. Firstly, taking the changing nature of career construction among youth in late modern societies, such as the trend towards sustainable farming linked to self-fulfillment, into consideration is important. Farm entry studies have documented findings on the strong emotional motivations of today’s youth that’s reflected in both their way of life and their involvement with attractive farming practices. For agricultural extension and rural communications specialists, career communications about the farming profession will need to encompass the motivational factors of *boundaryless*, *protean*, and *intrinsic* engagement in the careers of today’s youth and how their career initiations are formed in response to these motivations.

Secondly, highlighting new opportunities in the farming profession, like geographical flexibility (e.g., high-tech collaboration for farming practices in the scope of urban agriculture) or the innovation capacity of farming (e.g., alternative farming practices in the context of digital agriculture) for example, is important for increasing the awareness of the new image of the farming profession. For educational institutions, providing successful examples of “others” may help prepare students and provide them with more self-efficacy beliefs in farming careers.

Finally, as discussed by Lie and Servaes (2015), different from environmental communications, agricultural communications is often not concerned with specific target group communications activities. However, as argued by this review study, agricultural communications campaigns across digital platforms for targeted groups such as young females, or young people who are not in education, employment, or training (NEET) can play a role in changing the negative perceptions of a rural way of life and can help them to perceive farming as a long-term career option.

## Abbreviations

ICT: Information and Communication Technologies
LMS: Learning Management Systems
MOOCs: Massive Open Online Courses
NEETs: Not in Education, Employment, or Training
OERs: Open Education Repositories
PRISMA: Preferred Reporting Items for Systematic Reviews and Meta-Analysis Method
STEM: Science, Technology, Engineering, and Mathematics

## References

[CR1] Accesstoland. 2018. Europeʼs new farmers Innovative ways to enter farming and access land. https://www.accesstoland.eu/IMG/pdf/a2l_newentrants_handbook.pdf. Accessed 16 Feb 2022.

[CR2] Acuña TB, Monckton D, Boersma M, Bailey A, Gracie A (2019). Design and delivery of a masterclass in horticultural business. International Journal of Innovation in Science and Mathematics Education.

[CR3] Adro F, Franco M (2020). Rural and agri-entrepreneurial networks: A qualitative case study. Land Use Policy.

[CR4] Afere L, Oluwaseun A, Varun B, Courières CB, Mabonga L, Ocansey M, Neate P (2019). Making agriculture attractive to young people. Technical Centre for Agricultural and Rural Cooperation CTA.

[CR5] Agroop 2018. Study: young European farmers are few, highly qualified and invest a lot of money. https://medium.com/agroop/study-young-european-farmers-are-few-highly-qualified-and-invest-a-lot-of-money-ceb1b23ef02d. Accessed 09 Apr 2021.

[CR6] Ahearn, M., and D. Newton. 2009. Beginning Farmers and Ranchers. USDA Economic Research Service Bulletin Number 53.

[CR7] Akcaoglu M, Green LS (2019). Teaching systems thinking through game design. Educational Technology Research and Development.

[CR8] Araújo, S.O., R.S. Peres, J. Barata, F. Lidon, and J.C. Ramalho. 2021. Characterising the agriculture 4.0 landscape—emerging trends, challenges and opportunities. Agronomy 11.

[CR9] Armanda DT, Guinee JB, Tuckker A (2019). The second green revolution: Innovative urban agriculture's contribution to food security and sustainability—A review. Global Food Security.

[CR10] Arulchelvan P, Yunus MMd (2020). WHATSPEAK: Audiovisual digital assessment in enhancing confident and independent speaking skills. Universal Journal of Educational Research.

[CR11] Baker TT, Radel C, Dale-Hallett L, Forge C (2021). Photovoice, claiming visibility, and women's farming identities in Australia. Emotion, Space and Society.

[CR12] Ball JA (2020). Women farmers in developed countries: A literature review. Agriculture and Human Values.

[CR13] Barhate, B., and M. Dirani. 2021. Career aspirations of generation Z: a systematic literature review. European Journal of Training and Development, pp. 139–157.

[CR14] Benis K, Ferrão P (2018). Commercial farming within the urban built environment–taking stock of an evolving field in northern countries. Global Food Security.

[CR15] Bowen R, Morris W (2019). The digital divide: Implications for agribusiness and entrepreneurship. Lessons from Wales. Journal of Rural Studies.

[CR16] Boyle, K.A. 2022. Career identities and Millennials’ response to the graduate transition to work: Lessons learned. Journal of Education and Work, pp. 78–91.

[CR17] Brand, M., and E. Galdava. 2019. Engaging youth in agriculture through information and communication technologies. USAID. https://www.usaid.gov/sites/default/files/documents/15396/Feed-the-Future-CaseStudy-Youth-Ag-ICT.pdf. Accessed on 21 Feb 2022.

[CR18] Bright J, Pryor R, Wilkenfeld S, Earl J (2005). The role of social context and serendipitous events in career decision making. International Journal for Educational and Vocational Guidance.

[CR19] Broad GM (2020). Know your indoor farmer: square roots, techno-local food, and transparency as publicity. American Behavioral Scientist.

[CR20] Bruce AB (2019). Farm entry and persistence: Three pathways into alternative agriculture in southern Ohio. Journal of Rural Studies.

[CR21] Burnette JL, O’Boyle EH, VanEpps EM, Pollack JM, Finkel EJ (2013). Mind-sets matter: A meta-analytic review of implicit theories and self-regulation. Psychological Bulletin.

[CR22] Caskie P (2018). Human capital and the CAP: The case for radical policy reform. Euro Choices.

[CR23] Cole M, Stewart K (2017). ‘A new life in the countryside awaits’: Interactive lessons in the rural utopia in ‘farming’ simulation games. Discourse: Studies in the Cultural Politics of Education.

[CR24] Conway SF, Farrell M, McDonagh J, Kinsella A (2020). mobilising land mobility in the European Union: an under-researched phenomenon. International Journal of Agricultural Management.

[CR25] Daum T, Ygué Adegbola P, Adegbola C, Daudu C, Issa F, Kamau G, Oumar Kergna A, Mose L, Ndirpaya Y, Fatunbi O, Zossou R, Kirui O, Birner R (2022). Mechanization, digitalization, and rural youth—Stakeholder perceptions on three mega-topics for agricultural transformation in four African countries. Global Food Security.

[CR26] Deng X, Xu D, Zeng M, Qi Y (2019). Does Internet use help reduce rural cropland abandonment?. Evidence from China. Land Use Policy.

[CR27] Denton-Calabrese T, Mustain P, Geniets A, Hakimi L, Winters N (2021). Empowerment beyond skills: Computing and the enhancement of self-concept in the go_girl code+create program. Computers & Education.

[CR28] Dewey, C., 2017. A growing number of young Americans are leaving desk jobs to farm. Wash. Post. https://www.washingtonpost.com/business/economy/a-growing-number-of-young-americans-are-leaving-desk-jobs-to-farm/2017/11/23/e3c018ae-c64e-11e7-afe9-4f60b5a6c4a0_story.html?utm_term=.6f8f694fec02. Accessed 2 Feb 2022.

[CR29] Dias CS, Rodrigues RG (2019). Agricultural entrepreneurship and the financial crisis. Global Business and Economics Review.

[CR30] Dolci, P., and C. Perrin. 2018. Neo-farmers: Drivers of farming systems innovation and of the transition to agro-ecology? The case of Alentejo (Portugal). 13. European IFSA Symposium.

[CR31] Donnelly, M., 2014. Technology will allow for white-collared farmers. https://www.agriland.ie/farming-news/technology-will-allow-white-collared-farmers/. Accessed 02 Feb 2022.

[CR32] Drottberger A, Melin M, Lundgren L (2021). Alternative food networks in food system transition—values, motivation, and capacity building among Young Swedish Market Gardeners. Sustainability.

[CR33] Eistrup M, Sanches AR, Munoz-Rojas J, Pinto-Correia T (2019). A “Young Farmer Problem”? Opportunities and constraints for generational renewal in farm management. Land.

[CR34] Fairbairn M, Guthman J (2020). Agri-food tech discovers silver linings in the pandemic. Agriculture and Human Values.

[CR35] Farrell M, Murtagh A, Weir L, Conway SF, McDonagh J, Mahon M (2022). Irish organics, innovation and farm collaboration: A pathway to farm viability and generational renewal. Sustainability.

[CR36] Feinberg RM (2020). The new contadini: Transformative labor in Italian vineyards. Agriculture and Human Values.

[CR37] Fernández-Planells A, Orduna-Malea E, Feixa Pampols C (2021). Gangs and social media: A systematic literature review and an identification of future challenges, risks and recommendations. New Media & Society.

[CR38] Filimonova, N.G., M.G. Ozerova, I.N. Ermakova, and N.B. Miheeva. 2019. Crowdfunding as the way of projects financing in agribusiness. IOP Conference Series. Earth and Environmental Science 315.

[CR39] Flynn, P., A. Mujčinović, T. Ferreira, S. Bojnec, G. Neagu, I. Unay-Gailhard, A. Rocca, V. Lendzhova, and D. Bojadjieva. 2022 Challenges Associated with Formal Education in Rural Areas. Policy Brief Rural NEET Youth Network, COST Action CA18213. https: https://rnyobservatory.eu/web/wp-content/uploads/2022/04/Policy-Brief-Challenges-Education-Rural-Areas.pdf. Accessed 28 May 2022.

[CR40] Góngora R, Milán MJ, López-i-Gelats F (2019). Pathways of incorporation of young farmers into livestock farming. Land Use Policy.

[CR41] Guarín A, Rivera M, Pinto-Correia T, Guimar N, Šūmane S, Moreno-Pérez OMA (2020). A new typology of small farms in Europe. Global Food Security.

[CR42] Gul D, Demiryürek K (2020). Information and communication technologies usage in the rural and urban areas: The case of Ankara, Turkey. Anadolu Journal of Agricultural Sciences.

[CR43] Hackfort S (2020). Patterns of inequalities in digital agriculture: A systematic literature review. Sustainability.

[CR44] Hall DT (1996). The career is dead—Long live the career: A relational approach to careers (Jossey-Bass Business & Management Series).

[CR45] Hango D, Zarifa D, Pizarro Milian R, Seward B (2019). Roots and STEMs? Examining field of study choices among northern and rural youth in Canada. Studies in Higher Education.

[CR46] Hearn G, Collie N, Lyle P, Choi JHJ, Foth M (2014). Using communicative ecology theory to scope the emerging role of social media in the evolution of urban food systems. Futures.

[CR47] Hull CE, Hung YTC, Hair N, Perotti V, DeMartino R (2007). Taking advantage of digital opportunities: A typology of digital entrepreneurship. International Journal of Networking and Virtual Organizations.

[CR48] Ionitescu S, Correia de Melo RH, Popovici D, Conci A (2019). BIZ4FUN—3D virtual world as a motivator for youth entrepreneurship education. Scientific Papers: Management, Economic Engineering in Agriculture & Rural Development.

[CR49] Jimenez C (2017). From telephones in rural Oaxaca to mobile phones among Mixtec farm workers in Oxnard, California. New Media & Society.

[CR50] Jimenez IC, García L, Violante MG, Marcolin F, Vezzetti E (2021). Commonly Used external tam variables in e-learning, agriculture and virtual reality applications. Future Internet.

[CR51] Kamarudin K, Untari R, Rashid M (2020). Sustaining rural livelihood through entrepreneurship and creative village development: Malaysia and Indonesia experience. Scientific Papers: Management, Economic Engineering in Agriculture & Rural Development.

[CR52] Kawamorita H, Takahashi N, Demiryurek K (2020). Media literacy and rural women entrepreneurship: Experience from Japan and Turkey. Nordic Journal of Media Management.

[CR53] Klerkx L, Jakku E, Labarthe P (2019). A review of social science on digital agriculture, smart farming and agriculture 4.0: New contributions and a future research agenda. NJAS-Wageningen Journal of Life Sciences.

[CR54] Krumboltz JD (2009). The happenstance learning theory. Journal of Career Assessment.

[CR55] Lajoie-O'Malley A, Bronson K, van der Burg S, Klerkx L (2020). The future(s) of digital agriculture and sustainable food systems: An analysis of high-level policy documents. Ecosystem Services.

[CR56] Lans T, Seuneke P, Klerkx L, Carayannis E (2017). Agricultural entrepreneurship. Encyclopedia of creativity, invention, innovation and entrepreneurship.

[CR57] Lehberger M, Hirschauer N (2016). Recruitment problems and the shortage of junior corporate farm managers in Germany: The role of gender-specific assessments and life aspirations. Agriculture and Human Values.

[CR58] Lent RW, Brown SD, Hackett G (1994). Toward a unifying social cognitive theory of career and academic interest, choice, and performance [monograph]. Journal of Vocational Behavior.

[CR59] Lent RW, Brown SD, Hackett G (2000). Contextual supports and barriers to career choice: A social cognitive analysis. Journal of Counseling Psychology.

[CR60] Lenzi, D. 2021. Meeting young farmers´ambitions: A condition for the success of the new CAP. https://medium.com/ecajournal/meeting-young-farmers-ambitions-a-condition-for-the-success-of-the-new-cap-e630d9358508. Accessed 10 Feb 2022.

[CR61] Levoli C, Belliggiano A, Marandola D, Milone P, Ventura F (2019). Information and communication infrstructures and new business models in rural areas: The case of Molise region in Italy. European Countryside.

[CR62] Lie R, Servaes J (2015). Disciplines in the field of communication for development and social change. Communication Theory.

[CR63] Madianou M, Miller D (2018). Polymedia: Towards a new theory of digital media in interpersonal communication. Economic and Social Changes Journal.

[CR64] Mainiero LA, Sullivan SE (2006). The opt out revolt: Why people are leaving companies to create kaleidoscope careers.

[CR65] Manning, L. 2019. Most people don´t consider farming as a career path. Square roots’ next-gen farmer training is hoping to change that. https://agfundernews.com/most-people-dont-consider-farming-as-a-career-path-square-roots-next-gen-farmer-training-is-hoping-to-change-that.html#:~:text=Indoor%20farming%20startup%20Square%20Roots,systems%20business%20planning%20and%20marketing. Accessed 10 Feb 2022.

[CR66] Mardhiyyah YS, Rasyidi MA, Hidayah L (2020). Factors affecting crowdfunding investor number in agricultural projects: The dummy regression model. Journal of Management & Agribusiness.

[CR67] Maria K, Maria B, Andrea K (2021). Exploring actors, their constellations, and roles in digital agricultural innovations. Agricultural Systems.

[CR68] May D, Arancibia S, Behrendt K, Adams J (2019). Preventing young farmers from leaving the farm: Investigating the effectiveness of the young farmer payment using a behavioural approach. Land Use Policy.

[CR69] Meuwissen MP, Feindt PH, Spiegel A, Termeer CJ, Mathijs E, de Mey Y, Finger R, Balmann A, Wauters E, Urquhart J, Vigani M, Zawalinska K, Herrera H, Nicholas-Davies P, Hansson H, Paas W, Slijper T, Coopmans I, Vroege W, Ciechomska A, Accatino F, Kopainsky B, Poortvliet M, Candel JJL, Maye P, Severini S, Senni S, Soriano B, Lagerkvist CJ, Peneva M, Gavrilescu C, Reidsma P (2019). A framework to assess the resilience of farming systems. Agricultural Systems.

[CR70] Modestino Alicia S, Sugiyama Keimei, Ladge Jamie (2019). Careers in construction: An examination of the career narratives of young professionals and their emerging career self-concepts. Journal of Vocational Behavior.

[CR71] Moher D, Liberati A, Tetzlaff J, Altman DG (2009). Preferred reporting items for systematic reviews and meta-analyses: The PRISMA statement. BMJ.

[CR72] Newsome L (2021). Disrupted gender roles in Australian agriculture: First generation female farmers’ construction of farming identity. Agriculture and Human Values.

[CR73] Oliveira L, Cardoso EL (2020). Engaging stakeholders in traditional food products through dissemination of knowledge and innovation based in digital platforms. Future of Food Journal on Food, Agriculture and Society..

[CR74] Ong, T.W.Y. and Lioa, W. 2020. Agroecological Transitions: A Mathematical Perspective on a Transdisciplinary Problem. Frontiers in Sustainable Food Systems 4(91), 10.3389/fsufs.2020.00091.

[CR75] Paltaki A, Michailidis A (2020). Students' training needs towards precision agriculture. International Journal of Sustainable Agricultural Management and Informatics.

[CR76] Perez RDG, Sendra MJM, Lopez-i-Gelats F (2020). Strategies and drivers determining the incorporation of young farmers into the livestock sector. Journal of Rural Studies.

[CR77] Pilgeram R, Amos B (2015). Beyond “Inherit It or Marry It”: Exploring how women engaged in sustainable agriculture access farmland. Rural Sociology.

[CR78] Pindado E, Sánchez M, Verstegen JA, Lans T (2018). Searching for the entrepreneurs among new entrants in European Agriculture: The role of human and social capital. Land Use Policy.

[CR79] Pinkard N, Erete S, Martin CK, McKinney de Royston M (2017). Digital Youth Divas: Exploring narrative-driven curriculum to Spark Middle School Girls’ interest in computational activities. Journal of the Learning Sciences.

[CR80] Pinto M, Leite C (2020). Digital technologies in support of students learning in higher education: Literature review. Digital Education.

[CR81] Polanin N, Melendez M, Carleo J, Matthews J, Brumfield R, O’Neill B, Heckman J (2017). Social media: Cultivating peer-to-peer farm women networks in New Jersey. International Journal of Agricultural Science.

[CR82] Prause L, Hackfort S, Lindgren M (2020). Digitalization and the third food regime. Agriculture and Humain Values.

[CR83] Pryor RGL, Bright JEH (2014). The chaos theory of careers (CTC): Ten years on and only just begun. Australian Journal of Career Development.

[CR84] Raftery, I. 2011. New Food Culture, a Young Generation of Farmers Emerges. http://www.nytimes. com/2011/03/06/us/06farmers.html?_r=0. Accessed 01 May 2013.

[CR85] Reid J, Smith E, Iamsuk N, Miller J (2016). Balancing the equation: Mentoring first-year female STEM students at a regional university. International Journal of Innovation in Science and Mathematics Education.

[CR86] Rissing A, Inwood S, Stengel E (2021). The invisible labor and multidimensional impacts of negotiating childcare on farms. Agriculture and Human Values.

[CR87] Rojewski JW (1999). The role of chance in the career development of individuals with learning disabilities. Learning Disability Quarterly.

[CR88] Roman-Alcalá, A. 2013. In conversation with Severine von Tscharner Fleming, a young farmer and activist. http://sfaq.us/2013/07/in-conversation-with-severine-von-tscharner-fleming-a-young-farmer-and-activist/. Accessed 10 Feb 2022.

[CR89] Sa’uadi AN, Rahman SA (2019). An agricultural-related information elements and provision for Agripreneurship one-stop information portal. International Journal of Advanced Science and Technology.

[CR90] Salomone PR, Slaney RB (1981). The influence of chance and contingency factors on the vocational choice process of nonprofessional workers. Journal of Vocational Behavior.

[CR91] Sánchez JM, Rodríguez JP, Espitia HE (2020). Review of Artificial Intelligence Applied in Decision-Making Processes in Agricultural Public Policy. Processes.

[CR92] Saugeres L (2002). Of tractors and men: Masculinity, technology and power in a French farming community. Sociologia Ruralis.

[CR93] Savickas ML, Brown SD, Lent RW (2005). Career construction theory and practice. Career development and counseling putting theory and research to work.

[CR94] Schaffers H, Vartiainen M, Bus J (2020). Digital innovation and the future of work.

[CR95] Seymour M, Utter A (2021). Veganic farming in the United States: Farmer perceptions, motivations, and experiences. Agriculture and Human Values.

[CR96] Shortall S, McKee A, Sutherland LA (2020). The performance of occupational closure: The case of agriculture and gender. Sociologia Ruralis.

[CR97] Simões F, Unay-Gailhard I, Mujčinović A, Fernandes B (2021). How to foster rural sustainability through farming workforce rejuvenation? Looking into involuntary newcomers' spatial (im)mobilities. Sustainability.

[CR98] Slámová M, Kruse A, Belčáková I, Dreer J (2021). Old but not old fashioned: Agricultural landscapes as European heritage and basis for sustainable multifunctional farming to earn a living. Sustainability.

[CR99] Stevens TM, Aarts N, Termeer CJAM, Dewulf A (2016). Social media as a new playing field for the governance of agro-food sustainability. Current Opinion in Environmental Sustainability.

[CR100] Stringer LC, Fraser ED, Harris D, Lyon C, Pereira L, Ward CF, Simelton E (2020). Adaptation and development pathways for different types of farmers. Environmental Science & Policy.

[CR101] Su L, Peng Y, Kong R, Chen Q (2021). Impact of E-commerce adoption on farmers’ participation in the digital financial market: Evidence from Rural China. Journal of Theoretical and Applied Electronic Commerce Research.

[CR102] Sutherland LA (2020). The ‘desk-chair countryside’: Affect, authenticity and the rural idyll in a farming computer game. Journal of Rural Studies.

[CR103] Sutherland LA (2020). Virtualizing the ‘good life’: Reworking narratives of agrarianism and the rural idyll in a computer game. Agriculture and Human Values.

[CR104] Tsiaousi A, Partalidou M (2020). Female farmers in Greece: Looking beyond the statistics and into cultural–social characteristics. Outlook on Agriculture.

[CR105] Unay-Gailhard I, Bojnec S (2021). Gender and the environmental concerns of young farmers: Do young women farmers make a difference on family farms?. Journal of Rural Studies.

[CR106] Unay-Gailhard I, Simões F (2022). Becoming a young farmer in the digital age—an island perspective. Rural Sociology.

[CR107] Unay-Gailhard I, Bavorova M, Bednařiková Z, Ponkina E (2019). “I Don’t Want to Work in Agriculture!” the transition from agricultural education to the labor market in Rural Russia. Rural Sociology.

[CR108] Usman M, Sawaya A, Igarashi M, Gayman JJ, Dixit R (2021). Strained agricultural farming under the stress of youths’ career selection tendencies: a case study from Hokkaido (Japan). Humanities and Social Sciences Communications.

[CR109] Wilson S, Lyons T, Quinn F (2013). Should I stay or should I go? Rural and remote students in first year university STEM courses. Australian and International Journal of Rural Education.

[CR110] Yaghoubi Farani A, Karimi S, Izadi N, Ataei P (2019). Effect of virtual social networks on entrepreneurial behaviour of agriculture students in Iran. Applied Economics.

[CR111] Yan Z, Wang K, Wang Z-Y, Yu J, Tsai S-B, Li G (2018). Agricultural internet entrepreneurs’ social network behaviors and entrepreneurship financing performance. Sustainability.

[CR112] Yoon BK, Tae H, Jackman JA, Guha S, Kagan CR, Margenot AJ, Rowland DL, Weiss PS, Cho N-J (2021). Entrepreneurial talent building for 21st century agricultural innovation. ACS Nano.

